# From digital attitudes to online learning engagement: serial indirect associations through digital literacy and digital self-efficacy

**DOI:** 10.3389/fpsyg.2026.1943651

**Published:** 2026-10-08

**Authors:** Qing Liu, Yujie Wei, Rui Guo

**Affiliations:** 1 Research Office, Chongqing Industry and Information Vocational College, Chongqing, China; 2 Academic Affairs Office, Chongqing Wuyi Polytechnic, Chongqing, China; 3 School of Digital Economy and Intelligent Management, Chongqing Metropolitan College of Science and Technology, Chongqing, China

**Keywords:** digital attitude, digital literacy, digital self-efficacy, media psychology, online learning engagement, vocational students

## Abstract

**Background:**

Engagement with online learning depends not only on access to digital media but also on students' evaluations of these media, perceived competence, and confidence. This study examined the statistical indirect associations linking digital attitudes with online learning engagement through digital literacy and digital self-efficacy.

**Methods:**

A cross-sectional, anonymous survey was completed by 548 students from four vocational colleges in southwest China. A hierarchical measurement model was estimated from all 38 item-level responses: digital literacy and online engagement were specified as second-order constructs through three and four first-order dimensions, respectively, whereas digital attitude and digital self-efficacy were first-order constructs measured directly by their items. Control-adjusted indirect effects were evaluated with 5,000 bootstrap resamples.

**Results:**

In the latent-variable model, digital attitudes were positively associated with digital literacy (β = 0.792, *p* < 0.001) and online engagement (β = 0.263, *p* < 0.001). Digital literacy was strongly associated with digital self-efficacy (β = 0.906, *p* < 0.001), and both digital literacy (β = 0.330, *p* = 0.001) and digital self-efficacy (β = 0.369, *p* < 0.001) were associated with engagement. The attitude–self-efficacy path was not significant in the latent model. In the control-adjusted composite-score analysis, the very small self-efficacy-only indirect effect had a confidence-interval lower bound of 0.00005; given its inconsistency with the latent model, H7 was not regarded as robustly supported. Under the common-latent-factor sensitivity specification, the literacy-only interval narrowly excluded zero, whereas the serial interval included zero.

**Conclusion:**

The findings are consistent with indirect associations involving closely related competence and confidence perceptions, not with two clearly independent psychological mechanisms. Because the study was cross-sectional and relied on one self-report survey, the reported paths are associative rather than causal; exact-fit rejection, common-method risk, limited separation between digital literacy and self-efficacy, and sensitivity of the serial indirect effect also warrant caution.

## 1 Introduction

Online platforms, mobile applications, and other digital media increasingly structure how students encounter information, communicate with teachers and peers, and participate in learning. From a media-psychology perspective, access alone does not determine participation: students' evaluations of digital learning media, their perceived capability to use those media, and their confidence in completing technology-mediated tasks may shape engagement. Digital attitudes capture cognitive, affective, and behavioral evaluations of digital technologies, whereas online learning engagement reflects students' behavioral, cognitive, affective, and social investment in mediated learning activities. Understanding the psychological processes connecting these constructs is particularly relevant for vocational students, whose learning frequently combines practical training with online resources.

The concept of digital attitude builds on research concerning computer and Internet attitudes. It has been described as an evaluative orientation toward digital educational technologies (Novikova et al., 2023), technology acceptance (Yuen and Ma, 2008), and the perceived consequences of Internet use (Fishbein and Ajzen, 1975; Jackson et al., 2003). Digital attitudes may also reflect perceived expertise, complexity, and orientations toward mobile media (Stokburger-Sauer and Plank, 2014). In attitude–behavior frameworks, favorable evaluations increase readiness to perform a behavior, although behavior also depends on resources and perceived control (Fishbein and Ajzen, 1975). A positive digital attitude should therefore be associated with engagement, but its association may partly operate through students' perceived competence and confidence.

Online engagement includes behavioral, cognitive, affective, and social participation in learning activities (Balasooriya et al., 2018; Ifenthaler et al., 2018; Hoi and Le Hang, 2021). Although digital media can make learning more flexible, students differ substantially in how actively and meaningfully they participate. Digital divides also concern skills and effective use, not only physical access (Apostolidou, 2022; Robles Morales et al., 2016). Consequently, the psychological resources that help students translate a favorable orientation toward digital media into sustained participation require closer examination.

Digital literacy comprises the technical, cognitive, and social-emotional capabilities needed to use, evaluate, create, and communicate through digital media (Ng, 2012; Shen et al., 2013). These capabilities can enable students to navigate platforms, evaluate online information, solve technical problems, and collaborate with others. Digital literacy is associated with readiness for online learning and the capacity to participate purposefully in digital environments (Baroudi and Shaya, 2022; Getenet et al., 2024). It is therefore a plausible statistical link between favorable digital attitudes and engagement: students who value digital media may be more willing to develop relevant capabilities, and those capabilities may be associated with less friction during online learning.

Digital self-efficacy is students' confidence that they can use digital technologies to complete learning tasks and manage difficulties. The intended conceptual distinction is between a capability repertoire and a capability judgment: digital literacy concerns the knowledge and skills a student reports possessing across technical, cognitive, and social-emotional domains, whereas digital self-efficacy concerns the student's belief that those capabilities can be mobilized successfully when digital-learning demands or difficulties arise. Thus, two students reporting similar skills could differ in their confidence about applying those skills under challenge. Experience and stronger skills may provide mastery information that supports self-efficacy, which may in turn encourage persistence and participation (Iraola-Real et al., 2023). Context-specific self-efficacy has been associated with attitudes toward technology use (Bonanati and Buhl, 2022), and confidence may help students manage anxiety or setbacks in mediated learning.

Taken together, the literature suggests a serial competence–confidence pathway: favorable digital attitudes may be associated with greater digital literacy; literacy may be associated with stronger digital self-efficacy; and both may be associated with engagement. (Prior et al., 2016) reported flow-on associations among digital attitude, literacy, self-efficacy, and online learning behavior, but the measurement distinctiveness of literacy and efficacy and their specified sequential indirect associations remain uncertain, particularly among vocational-college students. This study therefore tested the following hypotheses in a cross-sectional sample: digital attitude would be positively associated with online engagement (H1), digital literacy (H2), and digital self-efficacy (H3); digital literacy (H4) and digital self-efficacy (H5) would be positively associated with engagement; the attitude–engagement association would show specific indirect effects through digital literacy (H6) and digital self-efficacy (H7); and a serial indirect effect would be observed through digital literacy followed by digital self-efficacy (H8). These hypotheses concern statistical associations and indirect effects, not a demonstrated temporal or causal process.

## 2 Materials and methods

### 2.1 Participants and procedure

Participants were recruited from four vocational colleges in southwest China, with recruitment seeking representation across gender, grade level, and subject area. The available survey export did not retain college or class identifiers, college-specific sample allocations, or the original sampling frame. We therefore do not characterize the procedure as stratified cluster sampling and could not estimate intraclass correlations or conduct cluster-robust or multilevel sensitivity analyses. Formal survey data collection commenced after completion of the pre-test, on April 23, 2024, and continued until April 2025. The primary eligibility criteria were prior participation in online learning and a correct response to an independent attention-check item instructing respondents to select “disagree” (coded 2). Audit of the original export confirmed that completion time was not used to construct the analyzed sample; the previous statement that responses had to exceed 1 min was inaccurate and has been removed. A total of 1,083 questionnaires were initially collected. Of these, 510 failed the attention check, leaving 573; among those 573, a further 25 reported no prior online-learning experience. The resulting 548 records exactly reproduced the original analytic file, corresponding to a retention rate of 50.6%. In this primary sample, completion time had a median of 157 s (interquartile range 112.75–222.25; range 49–2,262); 10 responses were below 60 s and one equalled 60 s. The final sample included 177 males (32.3%) and 371 females (67.7%); 377 first-year students (68.8%), 124 second-year students (22.6%), and 47 third-year students (8.6%); 183 students (33.4%) in science and engineering disciplines, 330 (60.2%) in humanities and social sciences, and 35 (6.4%) in arts disciplines. All surveys were conducted anonymously. Participants took part on an informed and voluntary basis and were informed that they could discontinue the questionnaire at any time without adverse consequences. No separate ethics approval or exemption document was issued before data collection began. After data collection, the Research Office of Chongqing Industry and Information Vocational College, Chongqing, China, issued a subsequent institutional determination that this anonymous, non-interventional, minimal-risk educational questionnaire study qualified for exemption from full ethical review (reference IRB-2026-0601, issued June 15, 2026). A separately signed written informed-consent form was not obtained; the Research Office subsequently determined that the study met the conditions for waiver of this requirement, and participants' voluntary continuation of the anonymous questionnaire was treated as their agreement to participate.

### 2.2 Measures

The instruments measured general attitudes, capabilities, confidence, and engagement concerning digital learning media. They did not measure exposure to, use of, or attitudes toward specific artificial-intelligence tools; accordingly, no AI-specific claims are tested in this study.

The instruments originated in English. An English-language academic at Xiamen University translated the items into Chinese, after which a Chinese-language academic at East China Normal University reviewed and refined the wording for clarity and natural expression. An educational-psychology professor then reviewed the questionnaire for conceptual and contextual appropriateness. No independent back-translation was conducted. The Chinese questionnaire was pre-tested from April 1 to April 22, 2024, in an independent sample of 493 undergraduate and vocational-college students who were not included in the 1,083 formal-survey responses or the final analytic sample. Formal survey data collection began after the pre-test was completed. The pre-test showed acceptable internal consistency for digital attitude (α = 0.848), digital literacy (α = 0.927), digital self-efficacy (α = 0.859), and online engagement (α = 0.936). The pre-test assessed item wording and internal consistency but did not include factor-analytic, convergent, discriminant, or criterion-related tests of construct validity. No items were removed or further revised after the pre-test.

For clarity, DA1–DA7 refer to the seven item-level indicators of digital attitude and DSE1–DSE5 to the five item-level indicators of digital self-efficacy. For the two hierarchical constructs, DL1–DL3 denote the first-order technical, cognitive, and social-emotional literacy factors, which are themselves measured by items b1–b6, b7–b8, and b9–b10, respectively. OE1–OE4 denote the first-order behavioral, cognitive, affective, and social engagement factors, each measured by four items (d1–d4, d5–d8, d9–d12, and d13–d16, respectively). Thus, DL1–DL3 and OE1–OE4 are latent first-order dimensions rather than dimension-level composite scores. Appendix A gives the wording of all 38 items, and Appendix B reports their standardized loadings in the hierarchical model.

#### 2.2.1 Digital attitude scale

The seven-item scale developed by Ng (2012) measured students' general attitudes toward information and communication technology. Each item was rated from 1 (strongly disagree) to 5 (strongly agree), with higher scores indicating more favorable digital attitudes. A sample item is “I like using digital technology to learn.” Internal consistency in this sample was α = 0.883.

#### 2.2.2 Digital literacy scale

Based on Ng (2012)'s scale, the digital literacy scale contains 10 items covering three dimensions: technical, cognitive, and social-emotional literacy. In this study, digital literacy refers to students' general ability to use, evaluate, and adapt to digital technologies in learning contexts. A 5-point Likert scale (1 = strongly disagree, 5 = strongly agree) was used, with higher scores indicating higher digital literacy. A sample item is: “I can easily learn new technologies.” The scale showed good internal consistency in the present sample (Cronbach's α = 0.950).

#### 2.2.3 Digital self-efficacy scale

The Generalized Self-Efficacy Scale (GSES) developed by Schwarzer and Jerusalem (1995) and Wang et al. (2001), and adapted into a 5-item digital-technology version for higher-education teachers by Wang and Chu (2023), was used. For administration to vocational-college students, teaching-related referents were replaced with corresponding study- and learning-related referents; Appendix A reports the exact wording administered. The independent pre-test provided evidence of internal consistency but no separate construct-validity evidence for this student-adapted version. A 5-point Likert scale (1 = strongly disagree, 5 = strongly agree) was adopted, with higher scores indicating stronger digital self-efficacy. A sample item is: “No matter what happens, I can easily handle various situations involving the use of digital technologies.” In this study, the Cronbach's α coefficient for this scale was 0.915, and the composite reliability (CR) was 0.916, indicating satisfactory internal consistency and composite reliability.

Both instruments nevertheless rely on self-reports rather than objective performance. An item-content audit showed that most digital-literacy items concern reported skills, information evaluation, or digital social practices, whereas the self-efficacy items emphasize coping, persistence, and successful task execution. However, literacy items such as “I can easily learn new technologies” and “I have good digital technology skills” are themselves self-evaluative, and the motivation wording of DL1-4 is not a pure performance indicator. These items may partly capture perceived confidence or motivation. We retained the validated scale specification, but this content overlap means that the operational distinction is less clear than the theoretical distinction and requires direct empirical testing and cautious interpretation.

#### 2.2.4 Online engagement scale

The 16-item scale by Wu (2023), developed by Hoi and Le Hang (2021), was used. It comprises four sub-dimensions: behavioral engagement (BE), cognitive engagement (CE), affective engagement (AE), and social engagement (SE). A 5-point Likert scale (1 = strongly disagree, 5 = strongly agree) was employed. Sample items include “I take notes when participating in online discussions” and “I like online learning activities.” The total scale showed good internal consistency in the present sample (Cronbach's α = 0.965).

### 2.3 Statistical analysis

All analyses were reproduced from the final 548-case data set in Python 3.12. Confirmatory factor analysis and structural equation modeling used semopy 2.3.11 with maximum-likelihood estimation, while descriptive statistics, internal-consistency estimates, Pearson correlations, and the control-adjusted serial-mediation regressions used pandas and statsmodels 0.14.6. The CFA and latent-variable SEM used the 38 individual questionnaire items as observed indicators; no dimension-level mean or composite score was substituted for an item in either model. Digital attitude was a first-order factor measured by DA1–DA7, and digital self-efficacy was a first-order factor measured by DSE1–DSE5. Digital literacy was a second-order factor indicated by the first-order technical (six items), cognitive (two items), and social-emotional (two items) factors. Online engagement was a second-order factor indicated by the first-order behavioral, cognitive, affective, and social engagement factors, each measured by four items. No correlated measurement errors were specified. Model fit was evaluated using exact χ^2^, χ^2^/*df*, CFI, TLI, NFI, RMSEA, and SRMR. A Bollen–Stine test with 1,000 re-estimated bootstrap samples evaluated exact fit (Bollen and Stine, 1992). Indirect effects were evaluated using 5,000 bootstrap resamples and percentile 95% confidence intervals. Gender, grade, and subject area were included as dummy-coded covariates in every composite-score mediation equation (reference groups: male, first year, and science/engineering, respectively). Because all focal variables came from the same cross-sectional self-report questionnaire, Harman's single-factor test was reported as a limited diagnostic of possible common-method variance, not as proof that bias was absent (Podsakoff et al., 2003). The heterotrait–monotrait ratio (HTMT) supplemented the Fornell–Larcker assessment of discriminant validity (Henseler et al., 2015).

As a sensitivity analysis, we also estimated a marker-free common latent factor model in which every item loaded on an additional method factor constrained to be orthogonal to all substantive factors; the substantive measurement and structural specification was otherwise unchanged. This diagnostic tests whether accounting for a shared item-level factor materially alters the structural estimates. The three specific indirect effects and their total were calculated from the unstandardized structural coefficients in this model and evaluated using 5,000 non-parametric percentile bootstrap resamples; all resamples converged without negative variance estimates. Standardized coefficient products were also calculated to describe relative magnitude. Because the questionnaire did not include a theoretically unrelated marker variable and the common-factor approach relies on strong identification assumptions, it was treated as a sensitivity analysis rather than as a definitive correction for common-method bias.

To evaluate the low loading of DA2, we re-estimated the CFA and latent-variable SEM after removing that item and recomputed the digital-attitude composite from the remaining six items. The control-adjusted indirect-effect analysis was then repeated with 5,000 bootstrap resamples using the same model, covariates, and random seed. We compared model fit, digital-attitude CR and AVE, standardized structural coefficients, explained variance, and the three specific indirect effects with the pre-specified seven-item results.

We also audited screening directly from the 1,083-record platform export. Because the originally analyzed 548 cases had not been filtered by completion time, the primary results retain that sample and a *post-hoc* sensitivity analysis evaluates stricter response-quality rules. The strict sample excluded responses completed in 60 s or less, invariant responses across all 38 substantive items, and later submissions from repeated non-guest user identifiers; the earliest eligible submission was retained. These non-overlapping rules excluded 74 records and produced *n* = 474. Generic guest identifiers and potentially shared institutional IP addresses prevented definitive duplicate identification for every record, so IP repetition alone was not treated as evidence of duplication. We re-estimated the latent-variable SEM and the 5,000-resample indirect-effect analysis in the strict sample. Pearson chi-square tests compared retained and excluded respondents on gender, grade, and subject area.

The serial indirect-effect model was estimated following the statistical logic of multiple mediation and conditional process analysis (Preacher and Hayes, 2008; Hayes, 2018). The directional specification was used to decompose the hypothesized indirect associations and should not be interpreted as establishing temporal order. In this model, digital attitude (*DA*) was specified as the independent variable, digital literacy (*DL*) and digital self-efficacy (*DSE*) as two mediators, and online engagement (*OE*) as the dependent variable. The structural relations can be expressed as:


$$\begin{array}{l} DL=a_{1}DA+e_{1}, \end{array}$$ (1a)

$$\begin{array}{l} DSE=a_{2}DA+d_{21}DL+e_{2}, \end{array}$$ (1b)

$$\begin{array}{l} OE=c^{\prime }DA+b_{1}DL+b_{2}DSE+e_{3}, \end{array}$$ (1c)

where *a*_1_, *a*_2_, *d*_21_, *b*_1_, *b*_2_, and *c*′ denote path coefficients, and *e*_1_, *e*_2_, and *e*_3_ denote residual terms. The three specific indirect effects were calculated as:


$$\begin{array}{l} Ind_{1}=a_{1}b_{1}, \end{array}$$ (2a)

$$\begin{array}{l} Ind_{2}=a_{2}b_{2}, \end{array}$$ (2b)

$$\begin{array}{l} Ind_{3}=a_{1}d_{21}b_{2}. \end{array}$$ (2c)

Here, *Ind*_1_ represents the path *DA* → *DL* → *OE*, *Ind*_2_ represents the path *DA* → *DSE* → *OE*, and *Ind*_3_ represents the serial path *DA* → *DL* → *DSE* → *OE*. The total indirect effect was calculated as:


$$\begin{array}{l} \text{Total indirect effect}=Ind_{1}+Ind_{2}+Ind_{3}. \end{array}$$ (3)

Equations 1a–c specify the structural relations; Equations 2a–c define the three specific indirect effects; and Equation 3 defines their sum as the total indirect effect. Percentile Bootstrap confidence intervals were used to evaluate the significance of indirect effects. An indirect effect was considered significant when its 95% confidence interval did not include zero (Preacher and Hayes, 2008; Hayes, 2018).

## 3 Results

### 3.1 Screening audit and response-quality sensitivity

The raw-export audit reproduced the analytic sample exactly: 510 of 1,083 responses failed the attention check, and 25 of the 573 attention-check passes reported no online-learning experience, leaving 548. The previously described completion-time rule had not been applied. A time-only sensitivity analysis excluding the 11 responses completed in 60 s or less (*n* = 537) retained the direction and significance pattern of all six latent structural paths.

The stricter response-quality sensitivity sample (*n* = 474) additionally excluded 60 invariant response patterns and three later submissions associated with repeated non-guest user identifiers. Model fit was χ^2^ = 1903.613, *df* = 652, χ^2^/*df* = 2.920, CFI = 0.910, TLI = 0.903, RMSEA = 0.064, and SRMR = 0.047. The standardized paths remained positive and significant for attitude → literacy (β = 0.769, *p* < 0.001), literacy → self-efficacy (β = 0.874, *p* < 0.001), attitude → engagement (β = 0.350, *p* < 0.001), literacy → engagement (β = 0.241, *p* = 0.020), and self-efficacy → engagement (β = 0.361, *p* < 0.001); attitude → self-efficacy remained non-significant (β = 0.038, *p* = 0.475). The literacy-only indirect effect was 0.200, 95% CI [0.113, 0.300], and the serial indirect effect was 0.212, 95% CI [0.145, 0.283]. The self-efficacy-only effect was again inconclusive (0.033, 95% CI [−0.0067, 0.0817]). Thus, the principal conclusions did not depend on the flagged records.

Retention differed between the retained and excluded records by gender (χ^2^(1) = 36.850, *p* < 0.001, Cramer's *V* = 0.184) and subject area (χ^2^(2) = 10.855, *p* = 0.004, *V* = 0.100), but not by grade (χ^2^(2) = 1.109, *p* = 0.574, *V* = 0.032). These differences indicate that selection into the analytic sample was not demographically neutral.

### 3.2 Descriptive statistics and correlation analysis

The means, standard deviations, and Pearson correlation coefficients for each variable are shown in Table 1. Digital attitude, digital literacy, digital self-efficacy, and online engagement were all significantly and positively correlated with each other (*p* < 0.01), providing a basis for subsequent mediation effect analysis.

**Table 1:** Descriptive statistics and Pearson correlation analysis (*r*).

Construct	M	SD	Digital attitude	Digital literacy	Digital self-efficacy	Online engagement
Digital attitude	3.612	0.623	1			
Digital literacy	3.632	0.663	0.752	1		
Digital self-efficacy	3.636	0.678	0.685	0.860	1	
Online engagement	3.674	0.639	0.731	0.824	0.816	1

*M* = mean; *SD* = standard deviation. All correlations were significant at *p* < 0.01.

### 3.3 Reliability analysis

Cronbach's α coefficient was used to examine the internal consistency of each scale. As shown in Table 2, the α coefficients for digital attitude, digital literacy, digital self-efficacy, online engagement, and the total scale were 0.883, 0.950, 0.915, 0.965, and 0.979, respectively, all exceeding the acceptable criterion of 0.7. Moreover, deleting any item would not significantly increase the α coefficient, indicating that the scales have high reliability.

**Table 2:** Cronbach's α coefficients.

Construct	Cronbach's alpha	Items
Digital attitude	0.883	7
Digital literacy	0.950	10
Digital self-efficacy	0.915	5
Online engagement	0.965	16
Total	0.979	38

### 3.4 Validity analysis

Convergent validity was assessed using composite reliability (CR) and average variance extracted (AVE). Table 3 now distinguishes the item-to-first-order and first-order-to-second-order portions of the hierarchical model. For the two second-order constructs, the reported CR and AVE values were calculated from their first-order factor loadings; item-level loadings for the seven first-order dimensions are reported separately in Appendix B. The reported CR and AVE values exceeded 0.70 and 0.50, respectively. Item DA2 nevertheless had a low standardized loading (0.343), substantially below the commonly used 0.50 reference value. It was retained to preserve the content of the original scale, but results involving digital attitude should therefore be interpreted cautiously.

**Table 3:** Hierarchical measurement model and construct-level reliability.

Construct	Measurement level	Indicators	Std. loading range	CR	AVE
Digital attitude	Item → first order	DA1–DA7	0.343–0.871	0.894	0.559
Digital literacy	Item → first order	DL1: b1–b6; DL2: b7–b8; DL3: b9–b10	0.740–0.916	—	—
	First order → second order	DL1 (technical), DL2 (cognitive), DL3 (social-emotional)	0.941–0.967	0.966	0.904
Digital self-efficacy	Item → first order	DSE1–DSE5	0.801–0.850	0.916	0.685
Online engagement	Item → first order	OE1: d1–d4; OE2: d5–d8; OE3: d9–d12; OE4: d13–d16	0.723–0.891	—	—
	First order → second order	OE1 (behavioral), OE2 (cognitive), OE3 (affective), OE4 (social)	0.921–0.943	0.963	0.868

All 38 individual questionnaire items were entered as observed indicators. DL1–DL3 and OE1–OE4 are latent first-order factors, not dimension-level composite scores. For the first-order digital-attitude and digital-self-efficacy constructs, CR and AVE were calculated from item loadings; for the two second-order constructs, CR and AVE were calculated from first-order factor loadings. Exact standardized loadings are reported in Appendix B. CR, composite reliability; AVE, average variance extracted.

Discriminant validity was assessed by comparing the square roots of AVEs with the latent correlations (Fornell and Larcker, 1981). Several correlations in Table 4 exceeded the corresponding square roots of AVE, most notably the digital-literacy–self-efficacy correlation (*r* = 0.932). The supplementary HTMT analysis led to the same conclusion: HTMT was 0.922 for digital literacy and self-efficacy, 0.861 for digital literacy and engagement, and 0.867 for self-efficacy and engagement. Table 5 provides the full competing-model results. The hypothesized four-construct hierarchical model fit significantly better than the three-construct model that merged digital literacy and self-efficacy (Δχ^2^ = 93.039, Δ*df* = 3, *p* < 0.001), and the single-factor model fit markedly worse. The comparison therefore argues against complete empirical collapse of literacy and self-efficacy. It does not, however, override the failed Fornell–Larcker comparison, HTMT = 0.922, or the content overlap in the self-reported measures. We consequently retain the constructs for theory-based, qualified comparison while treating their separate structural effects as weakly discriminated rather than as evidence of two clearly independent mechanisms.

**Table 4:** Discriminant validity test.

Construct	AVE	Online engagement	Digital self-efficacy	Digital literacy	Digital attitude
Online engagement	0.868	**0.931**			
Digital self-efficacy	0.685	0.874	**0.828**		
Digital literacy	0.904	0.882	0.932	**0.951**	
Digital attitude	0.559	0.801	0.751	0.792	**0.748**

Bold diagonal values are the square roots of AVE; lower-triangular values are latent-variable correlations from the confirmatory factor analysis.

**Table 5:** Competing measurement models for construct distinctiveness.

Model	χ^2^	*df*	χ^2^/*df*	CFI	TLI	RMSEA	SRMR	*Δχ*^2^ (*Δdf*)
Four-construct hierarchical	2186.310	652	3.353	0.921	0.915	0.066	0.042	Reference
DL and DSE merged	2279.349	655	3.480	0.917	0.911	0.067	0.043	93.039 (3)^***^
Single factor	4676.705	665	7.033	0.795	0.783	0.105	0.057	–

DL, digital literacy; DSE, digital self-efficacy. The likelihood-ratio comparison is reported for the nested merged model. The single-factor model is included as a broad descriptive alternative. ^***^p < 0.001.

The DA2 deletion sensitivity results are summarized in Table 6. Removing DA2 increased digital-attitude CR from 0.894 to 0.911 and AVE from 0.559 to 0.632, and the remaining loadings ranged from 0.714 to 0.872. Overall fit did not improve: CFI and TLI were essentially unchanged, while χ^2^/*df*, RMSEA, and SRMR were slightly higher. All six standardized structural coefficients changed by no more than 0.003, and engagement *R*^2^ remained 0.824. The literacy-only and serial bootstrap intervals continued to exclude zero. The small self-efficacy-only effect became 0.034, 95% CI [−0.00007, 0.076], further supporting the decision not to regard H7 as robustly supported. Thus, deleting DA2 improves the digital-attitude reliability and convergence estimates but does not alter the substantive conclusions.

**Table 6:** Sensitivity analysis excluding the low-loading DA2 item.

Statistic	Seven-item digital attitude	DA2 excluded
CFA χ^2^ (*df*)	2186.310 (652)	2154.053 (616)
χ^2^/*df*	3.353	3.497
CFI / TLI	0.921 / 0.915	0.921 / 0.915
RMSEA / SRMR	0.066 / 0.042	0.068 / 0.042
Digital-attitude CR / AVE	0.894 / 0.559	0.911 / 0.632
Online-engagement *R*^2^	0.824	0.824
Largest absolute change in structural β	Reference	0.002
*Ind*_1_: DA → DL → OE	0.251 [0.157, 0.351]	0.228 [0.135, 0.325]
*Ind*_2_: DA → DSE → OE	0.035 [0.00005, 0.080]	0.034 [−0.00007, 0.076]
*Ind*_3_: DA → DL → DSE → OE	0.236 [0.166, 0.305]	0.223 [0.157, 0.289]
Total indirect effect	0.523 [0.435, 0.608]	0.485 [0.402, 0.564]

Indirect effects are unstandardized estimates with 95% percentile bootstrap confidence intervals based on 5,000 resamples. DA, digital attitude; DL, digital literacy; DSE, digital self-efficacy; OE, online engagement.

To further examine possible common method bias, Harman's single-factor test was conducted using all measurement items. The first unrotated factor accounted for 57.51% of the total variance, which is above the commonly used 50% reference value. This result suggests that common method bias may be present because all data were collected through a single self-report survey at one time point. Therefore, the findings should be interpreted with appropriate caution.

The marker-free common latent factor sensitivity model converged without negative variance estimates and showed χ^2^ = 1712.377, *df* = 614, χ^2^/*df* = 2.789, CFI = 0.944, TLI = 0.936, RMSEA = 0.057, and SRMR = 0.036. Standardized method-factor loadings ranged from 0.231 to 0.826 (median absolute loading = 0.555), indicating substantial shared item variance. The principal structural pattern remained: attitude was associated with literacy (β = 0.664, *p* < 0.001) and engagement (β = 0.338, *p* < 0.001); literacy was associated with self-efficacy (β = 0.856, *p* < 0.001) and engagement (β = 0.390, *p* < 0.001); and self-efficacy remained associated with engagement but was attenuated (β = 0.185, *p* = 0.038). The attitude–self-efficacy path remained non-significant (β = 0.026, *p* = 0.553). Engagement *R*^2^ decreased from 0.824 to 0.675. The common-factor indirect-effect estimates (unstandardized; standardized product in parentheses) were 0.202 (0.259), 95% bootstrap CI [0.001, 0.500], for the literacy-only path; 0.004 (0.005), 95% CI [−0.021, 0.063], for the self-efficacy-only path; and 0.082 (0.105), 95% CI [−0.045, 0.357], for the serial path. The total indirect effect was 0.288 (standardized-product sum 0.369), 95% CI [0.002, 0.666]. Thus, although the literacy-only interval narrowly excluded zero, the serial and self-efficacy-only intervals included zero. The diagnostic preserves the direction of the main point estimates but indicates that the serial indirect result, effect magnitudes, and original model's explanatory power are sensitive to shared-method adjustment.

### 3.5 Model fit testing

The prespecified hierarchical model was estimated directly from all 38 item-level responses. The 10 digital-literacy items and 16 online-engagement items loaded on their respective first-order dimensions, which in turn loaded on the two second-order constructs; dimension-level composite scores were not used in the CFA or latent-variable SEM. The reference values in Table 7 are commonly used heuristic guidelines for descriptive assessment rather than rigid or universally applicable cutoffs (Hair et al., 2009); interpretation therefore considers the indices jointly, model complexity, and the distinction between exact and approximate fit. As shown in Table 7, RMSEA, SRMR, CFI, and TLI indicated acceptable approximate fit, whereas χ^2^/*df* was above 3 and NFI was marginally below 0.90. The exact chi-square test was significant. In 1,000 Bollen–Stine bootstrap samples, none produced a chi-square value as large as the observed value; with the finite-sample correction, *p* = 0.001. Thus, exact fit was rejected even though several approximate-fit indices were acceptable. This mixed evidence is reported rather than replacing it with an undocumented *post-hoc* modified model.

**Table 7:** Model fit indices.

Index	Value	Reference value	Interpretation
χ^2^	2186.298 (*df* = 652), *p* < 0.001	Non-significant desirable	Exact fit rejected
χ^2^/*df*	3.353	<3	Above reference
RMSEA	0.066	<0.08	Acceptable
SRMR	0.042	<0.08	Acceptable
NFI	0.892	≥0.90	Marginally below
TLI	0.915	≥0.90	Acceptable
CFI	0.921	≥0.90	Acceptable
Bollen–Stine *p*	0.001 (1,000 resamples)	>0.05 desirable	Exact fit rejected

Reference values are commonly used heuristic guidelines, not universal cutoffs. RMSEA, root mean square error of approximation; SRMR, standardized root mean square residual; NFI, normed fit index; TLI, Tucker–Lewis index; CFI, comparative fit index. No correlated residual/error terms were estimated.

### 3.6 Structural model validation

Figure 1 illustrates the re-estimated structural model, and Table 8 reports the structural paths. Digital attitude was positively associated with digital literacy (β = 0.792, *p* < 0.001), consistent with H2, and the model accounted for 62.8% of its variance. Digital literacy was strongly associated with digital self-efficacy (β = 0.906, *p* < 0.001). The direct attitude–self-efficacy path was not significant (β = 0.033, *p* = 0.467), so H3 was not supported in the latent model. Digital self-efficacy (β = 0.369, *p* < 0.001), digital literacy (β = 0.330, *p* = 0.001), and digital attitude (β = 0.263, *p* < 0.001) were positively associated with online engagement, consistent with H5, H4, and H1, respectively. The model accounted for 82.4% of engagement variance. This high in-sample *R*^2^ should not be interpreted as independent predictive performance because the predictors and outcome were measured concurrently by the same self-report questionnaire and the predictors showed substantial empirical overlap.

**Figure 1:**
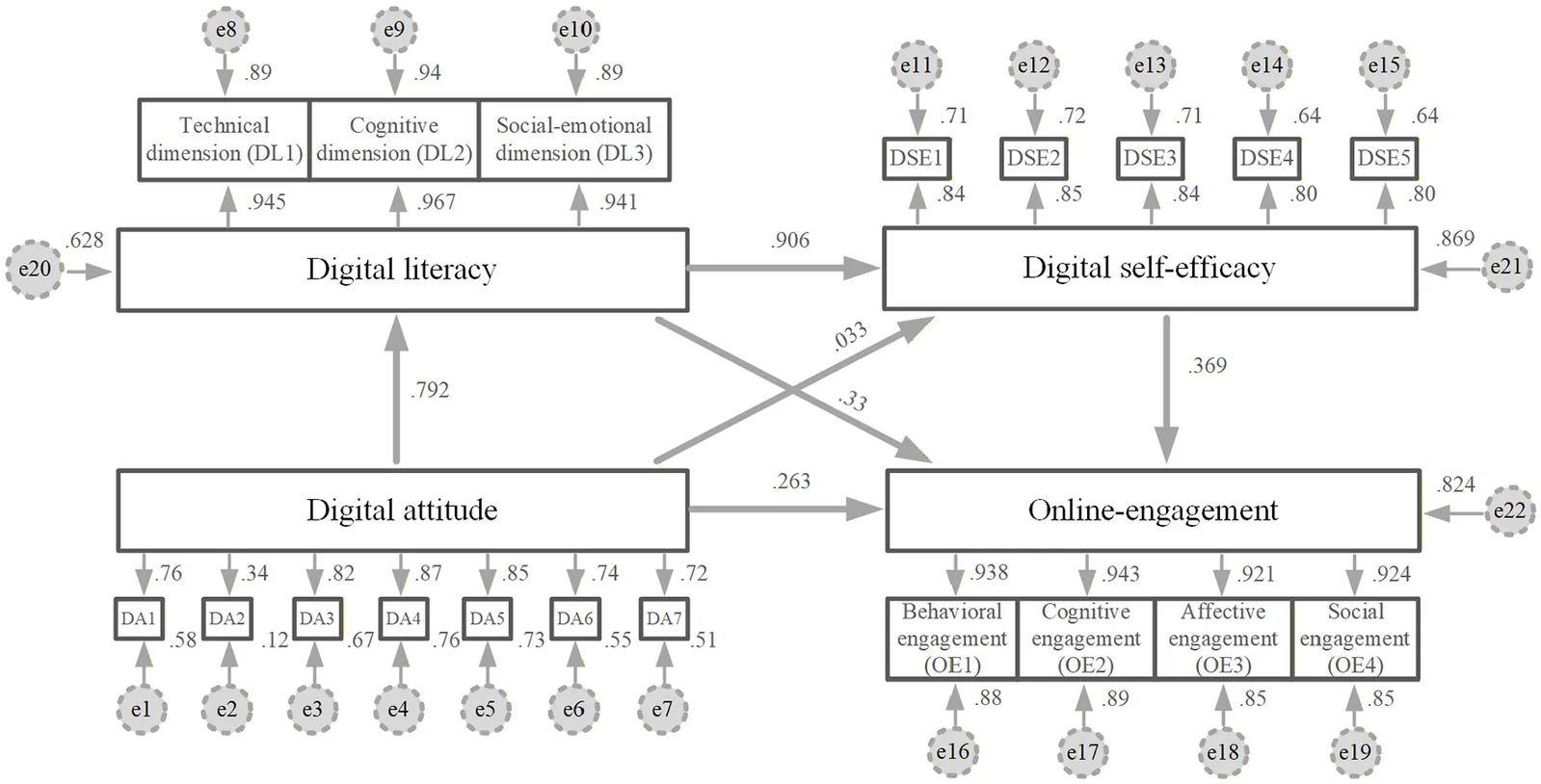
Hierarchical structural model estimated from all 38 item-level indicators. DA1–DA7 and DSE1–DSE5 are observed item indicators. DL1–DL3 and OE1–OE4 are latent first-order dimensions, not composite scores; their 10 digital-literacy items (b1–b10) and 16 online-engagement items (d1–d16) were included in estimation but are omitted from the diagram solely for readability. Values shown from the second-order factors to DL1–DL3 and OE1–OE4 are standardized second-order loadings. Other displayed values are standardized structural coefficients or item loadings; squared multiple correlations are shown beside displayed measurement indicators, and *R*^2^ is shown for endogenous constructs. No correlated measurement errors were specified.

**Table 8:** Structural model path analysis results.

Route			Unstd.	S.E.	C.R.	*p*	Std.(β)	*R* ^2^
Predictor	Direction	Outcome						
Digital attitude	→	Digital literacy	0.779	0.051	15.227	<0.001	0.792	0.628
Digital literacy	→	Digital self-efficacy	0.956	0.065	14.799	<0.001	0.906	0.869
Digital attitude	→	Digital self-efficacy	0.034	0.046	0.728	0.467	0.033	0.869
Digital self-efficacy	→	Online engagement	0.372	0.093	3.995	<0.001	0.369	0.824
Digital literacy	→	Online engagement	0.350	0.107	3.263	0.001	0.330	0.824
Digital attitude	→	Online engagement	0.274	0.046	5.957	<0.001	0.263	0.824

Unstd., unstandardized estimate; S.E., standard error; C.R., critical ratio; Std., standardized coefficient.

### 3.7 Testing of serial indirect effects

The serial indirect-effect model was re-estimated from composite scores with gender, grade, and subject area entered as covariates in all three equations. The total indirect effect was 0.523, with a 5,000-resample bootstrap 95% confidence interval of [0.435, 0.608]. The control-adjusted standardized regression coefficients were 0.755 for attitude → literacy, 0.090 for attitude → self-efficacy, 0.794 for literacy → self-efficacy, 0.224 for attitude → engagement, 0.324 for literacy → engagement, and 0.384 for self-efficacy → engagement. Grade 2 had a small negative coefficient in the engagement equation (β = −0.045, *p* = 0.050); all other demographic coefficients were non-significant. The indirect effects are shown in Figure 2.

**Figure 2:**
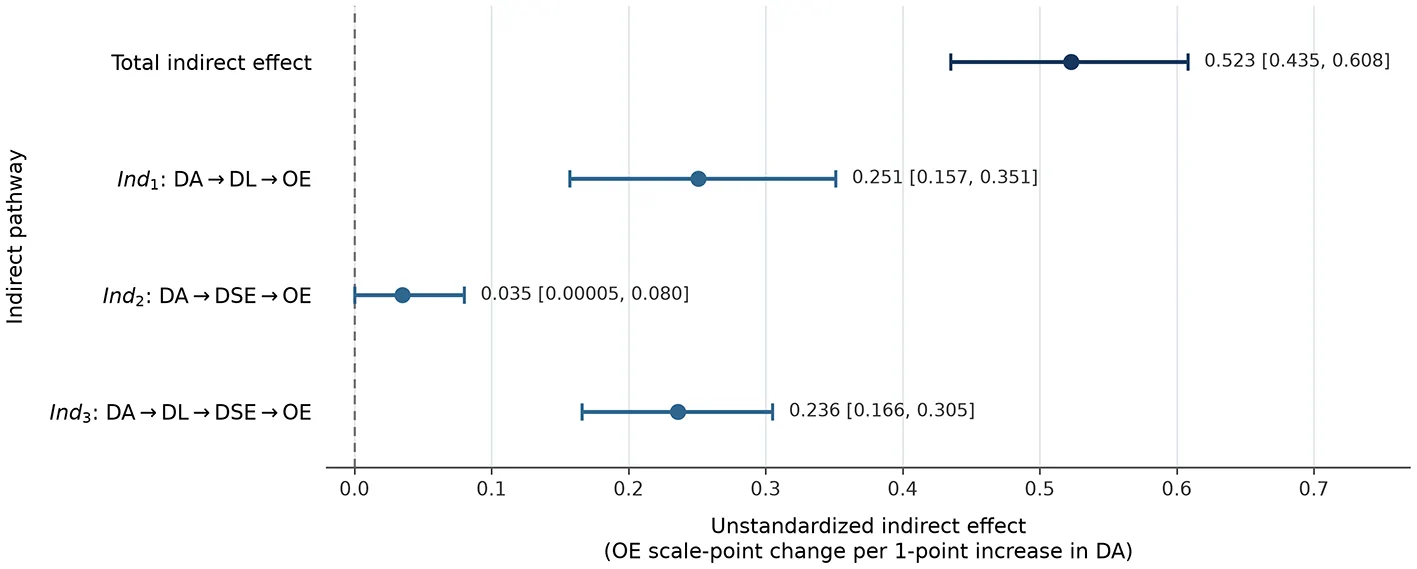
Bootstrap estimates of the total and specific indirect effects. Points represent unstandardized indirect-effect estimates, and horizontal bars represent 95% percentile bootstrap confidence intervals based on 5,000 resamples. The dashed vertical line indicates zero. The total indirect effect is the sum of the three specific indirect pathways. *Ind*_1_ refers to digital attitude → digital literacy → online engagement; *Ind*_2_ refers to digital attitude → digital self-efficacy → online engagement; and *Ind*_3_ refers to digital attitude → digital literacy → digital self-efficacy → online engagement.

All three percentile confidence intervals excluded zero: *Ind*_1_ = 0.251, 95% CI [0.157, 0.351]; *Ind*_2_ = 0.035, 95% CI [0.00005, 0.080]; and *Ind*_3_ = 0.236, 95% CI [0.166, 0.305]. H6 and H8 were therefore supported. H7 was not regarded as supported: although the composite-score interval for *Ind*_2_ narrowly excluded zero, its lower bound was only 0.00005, the effect was small, and the corresponding attitude–self-efficacy path was non-significant in the latent-variable SEM (β = 0.033, *p* = 0.467). This estimator-sensitive discrepancy makes the self-efficacy-only pathway inconclusive rather than independently supported. These cross-sectional indirect effects are consistent with, but do not establish, the proposed temporal sequence.

## 4 Discussion

### 4.1 Principal findings

This study examined psychological pathways connecting attitudes toward digital learning media with online engagement. The latent-variable results supported H1, H2, H4, and H5: favorable digital attitudes were associated with literacy and engagement, and both literacy and self-efficacy were associated with engagement. H3 was not supported in the latent model because digital attitude had no unique association with self-efficacy after digital literacy was included. The control-adjusted composite-score analysis supported the literacy-only (H6) and serial literacy–self-efficacy (H8) indirect paths. H7 was not counted as supported because its very small composite-score indirect effect was inconsistent with the non-significant attitude–self-efficacy path in the latent-variable model. In the common-latent-factor sensitivity model, the literacy-only interval only narrowly excluded zero and the serial interval included zero; H8 should therefore be understood as supported in the primary composite analysis but not robust to shared-method adjustment.

The pattern is consistent with a qualified competence–confidence account involving closely related self-perceptions rather than clearly independent psychological mechanisms. Favorable evaluations of digital media may accompany greater willingness to learn how to use those media, while perceived competence may supply the mastery basis for confidence. Confidence, in turn, is associated with participation and persistence. The weak and estimator-sensitive attitude–self-efficacy result suggests that positive evaluations alone may not reliably translate into confidence when competence is considered. However, temporal ordering cannot be verified with cross-sectional data, and reciprocal relations are plausible: engagement may also strengthen literacy, efficacy, and attitudes.

The DA2 sensitivity analysis shows that the primary pattern did not depend on the low-loading item: removing DA2 left model fit and all structural paths essentially unchanged, H6 and H8 remained non-zero in that item-deletion analysis, and H7 became clearly non-significant. This item-deletion result does not imply robustness to the separate common-method adjustment, under which the H8 interval included zero. We retained DA2 in the prespecified model to preserve the content and comparability of the original seven-item scale and to avoid a result-driven *post-hoc* deletion. The improved CR and AVE after deletion nevertheless show that the digital-attitude instrument requires revalidation, and future work should revise or replace DA2 prospectively.

In the primary composite-score analysis, digital literacy had the largest specific indirect-effect estimate, closely followed by the serial effect. The distinction between literacy as a reported capability repertoire and self-efficacy as confidence in mobilizing that repertoire is theoretically meaningful, and the merged model fit significantly worse. Nevertheless, the latent correlation of 0.932, HTMT of 0.922, self-evaluative wording in several literacy items, and the literacy–self-efficacy coefficient of 0.906 show that the measures do not achieve strong empirical separation. The serial estimate may therefore reflect an ordering among closely related competence perceptions rather than two clearly independent mechanisms. The model comparison supports retaining a qualified distinction; it does not establish satisfactory discriminant validity. Replication with refined measures, objective skill indicators, and longitudinal or experimental designs is needed.

The *R*^2^ of 82.4% for online engagement is unusually high for a cross-sectional behavioral model and should therefore be interpreted cautiously. Digital literacy, digital self-efficacy, and engagement were strongly correlated, and all focal constructs were measured in the same questionnaire at the same time. Construct overlap and shared method variance may consequently have inflated the covariance attributed to the structural predictors. Consistent with this concern, engagement *R*^2^ fell to 0.675 and the self-efficacy–engagement coefficient was approximately halved in the common latent factor sensitivity model. The common-factor bootstrap interval retained only narrow evidence for the literacy-only effect and included zero for the serial effect, further showing that the principal indirect-effect magnitudes and H8 interpretation are sensitive to the treatment of shared method variance. The reported *R*^2^ is an in-sample, model-dependent estimate and should not be interpreted as evidence that the model would achieve comparable predictive accuracy in an independent sample.

### 4.2 Theoretical implications

The findings extend research on digital-media use by examining evaluative processes together with closely related competence and confidence perceptions in one model. They do not establish digital literacy and digital self-efficacy as independent mechanisms; instead, the model describes their qualified joint association with engagement under weak empirical separation. They also emphasize that engagement is not simply a consequence of access to technology. Students' psychological relationship with digital media—what they think of the media, what they believe they can do with them, and how confident they feel when difficulties arise—is associated with how they participate in online learning. The vocational-college sample broadens the empirical setting, while the transparent non-significant pathway helps delimit the proposed sequence.

### 4.3 Practical implications

The results suggest several concrete, testable practices for vocational educators and institutions. To support digital attitudes, instructors can demonstrate how specific platforms and tools solve authentic vocational problems, provide short low-stakes orientation activities before graded online work, and use examples tied to students' trades or professional fields. To strengthen digital literacy, institutions can diagnose technical, information-evaluation, and online-collaboration needs at course entry and offer modular workshops, guided platform practice, troubleshooting resources, and peer mentoring targeted to the identified gaps. To support self-efficacy, educators can sequence tasks from simple to complex, model successful strategies, provide timely process-focused feedback, and ensure accessible help channels so that students accumulate credible mastery experiences rather than receiving encouragement alone. Online courses can then translate these supports into engagement through clear weekly participation routines, applied problem-solving tasks, structured reflection, and purposeful peer discussion. Where platform analytics are available, low participation can trigger early supportive contact, but such monitoring should be transparent and privacy-conscious. Because the present data are observational, these recommendations are hypotheses for intervention design rather than practices demonstrated to cause higher engagement; institutions should evaluate them with longitudinal indicators, platform behavior, and controlled or phased implementation.

### 4.4 Limitations and future research

Several limitations constrain interpretation. First, the cross-sectional design cannot establish temporal order or causality, even though the mediation model specifies directional paths. Longitudinal, cross-lagged, or experimental research is required. Second, all focal variables were collected in one self-report questionnaire. Harman's test and the marker-free common latent factor sensitivity analysis both indicate appreciable common-method risk, and neither diagnostic can establish that the bias has been removed. The questionnaire contained no theoretically unrelated marker variable, while the latent-method-factor diagnostic depends on restrictive orthogonality and loading assumptions. Future studies should separate measurements over time and combine surveys with platform logs, task performance, marker variables, or reports from other sources. Third, digital literacy and self-efficacy showed weak discriminant separation, so refined items and competing measurement models are needed. Fourth, DA2 had a low loading. Although its removal did not alter the structural conclusions, it improved digital-attitude CR and AVE; the item and scale should therefore be revalidated prospectively. Fifth, only 548 of 1,083 initial questionnaires were retained, and retention differed by gender and subject area. Although the time-only and strict response-quality sensitivity analyses preserved the main structural conclusions, the completion-time criterion had not been applied to the primary sample, the quality checks were necessarily *post hoc*, and generic guest identifiers prevented comprehensive duplicate detection. Selection and response-quality bias therefore remain possible. Sixth, college and class identifiers were not retained, so possible dependence among students recruited from the same institution or class could not be quantified or addressed with cluster-robust or multilevel analyses. Seventh, the sample came from four vocational colleges in southwest China and was predominantly female and first-year, limiting generalizability. Eighth, the instrument-adaptation procedure did not include independent back-translation, and the pre-test included both undergraduate and vocational-college students rather than only the target population. Moreover, beyond expert review and internal-consistency assessment, the pre-test did not provide independent factor-analytic, convergent, discriminant, or criterion-related evidence for the construct validity of the student-adapted digital self-efficacy scale. Future studies should use independent forward and backward translation, target-population cognitive interviewing, and prospective validation of the adapted scale. Finally, the measures concerned digital learning generally and did not assess any specific artificial-intelligence system; the findings must not be interpreted as evidence about AI acceptance or AI-supported learning.

## 5 Conclusion

Digital attitudes showed a direct association with online engagement and statistical indirect associations through digital literacy and the specified literacy–self-efficacy sequence in the primary composite analysis. The serial indirect effect was not robust under the common-latent-factor sensitivity specification. These cross-sectional results describe associations among closely related competence and confidence perceptions; they neither establish a temporal mediation process nor demonstrate two clearly independent psychological mechanisms. The failure to obtain robust support for H7, the method-sensitive evidence for H8, the high and potentially inflated engagement *R*^2^, rejection of exact model fit, limited discriminant separation, common-method risk, and cross-sectional design call for cautious interpretation and independent replication.

## Data Availability

The raw data supporting the conclusions of this article will be made available by the authors, without undue reservation.
