# Supplementary material for: From digital attitudes to online learning engagement: serial indirect associations through digital literacy and digital self-efficacy
Source: Front Psychol. 2026 Oct 8;17:1943651. doi: 10.3389/fpsyg.2026.1943651 (PMC13687038; doi:10.3389/fpsyg.2026.1943651)
Supplement: Supplementary file 1 [file Supplementary_file_1.zip › logo1-eps-converted-to.pdf]

frontiers
