# Supplementary material for: From digital attitudes to online learning engagement: serial indirect associations through digital literacy and digital self-efficacy
Source: Front Psychol. 2026 Oct 8;17:1943651. doi: 10.3389/fpsyg.2026.1943651 (PMC13687038; doi:10.3389/fpsyg.2026.1943651)
Supplement: Supplementary file 1 [file Supplementary_file_1.zip › Supplementary_Material.pdf]

Supplementary Material

1 SUPPLEMENTARY APPENDIX A. MEASUREMENT ITEMS

The items below reproduce, in English translation, the wording administered in the Chinese questionnaire. They were translated and linguistically reviewed from the cited source instruments as described in the Measures section of the main article. For the digital self-efficacy items, teaching-related referents in the source adaptation were replaced with study- and learning-related referents for vocational-college students. No other items were removed or revised after the pretest.

Table S1: Measurement items used in the questionnaire.

Code	Item wording
DA1	I like using digital technology to learn.
DA2	Using digital technology helps me learn better than not using it.
DA3	Digital technology makes learning more interesting.
DA4	Digital technology makes me more motivated to learn.
DA5	Digital technology makes me an autonomous and independent learner.
DA6	Learning with mobile technologies, such as smartphones, laptops, and tablets, has great potential.
DA7	Teachers should use more digital technology in classroom teaching.
DL1-1	I know how to solve digital technology problems that I encounter.
DL1-2	I can easily learn new digital technologies.
DL1-3	I can keep up with the latest developments in digital technology.
DL1-4	Mastering digital technology makes me more motivated to learn.
DL1-5	I have certain digital technology skills for creating and presenting work, such as presentations or blogs.
DL1-6	I have good digital technology skills.
DL2-1	I am confident in my ability to obtain and evaluate information from the Internet.
DL2-2	I am familiar with issues related to online activities, such as searching, cybersecurity, and intellectual property.
DL3-1	Digital technology enables me to collaborate better with peers in work or study.
DL3-2	I often receive help from online friends, such as through WeChat, Weibo, or Rednote.
DSE1	If I work hard enough, I can acquire digital technology skills.
DSE2	If I have enough time, I can use digital technology to complete my studies effectively.
DSE3	If I can seek help online, I can overcome digital technology difficulties encountered in learning.
DSE4	Even without help from others, I can independently overcome digital technology difficulties encountered in learning.
DSE5	No matter what happens, I can easily handle various situations involving the use of digital technologies.
OE1-1	I take notes when participating in online learning discussions.
OE1-2	I can stay focused during online learning activities.
OE1-3	Even when offline, I talk about topics related to online learning.
OE1-4	I have completed all online learning tasks on time.
OE2-1	Before participating in online learning discussions, I first browse the learning materials.
OE2-2	I try to connect what I learn in online learning activities with what I have learned before.
OE2-3	During online learning, I try to find additional online resources to understand difficult concepts.
OE2-4	I am motivated to improve my online learning skills.
OE3-1	I like online learning activities.
OE3-2	I look forward to participating in online learning activities.
OE3-3	I feel comfortable participating in online learning discussions.
OE3-4	I try to understand whether I have made mistakes during online learning.
OE4-1	I share learning materials with online classmates.
OE4-2	I often have new ideas in online learning discussions.
OE4-3	When I encounter problems in online learning, I ask the teacher for help.
OE4-4	I answer classmates' questions in online learning discussion forums.

2 SUPPLEMENTARY APPENDIX B. STANDARDIZED LOADINGS IN THE HIERARCHICAL MEASUREMENT MODEL

Supplementary Table S2 reports both levels of the measurement model. DA and DSE items load directly on their first-order constructs. The DL and OE items load on their respective first-order dimensions, and those dimensions subsequently load on the second-order DL and OE constructs. No dimension-level composite score was used as an observed indicator.

Table S2. Standardized loadings for all levels of the hierarchical measurement model.

Factor (level)	Indicator	Standardized loading	Indicator	Standardized loading	Indicator	Standardized loading	Indicator	Standardized loading
Digital attitude (first order)	DA1	0.764	DA2	0.343	DA3	0.817	DA4	0.871
	DA5	0.852	DA6	0.740	DA7	0.715		
Technical literacy, DL1 (first order)	DL1-1	0.768	DL1-2	0.845	DL1-3	0.865	DL1-4	0.839
	DL1-5	0.828	DL1-6	0.854				
Cognitive literacy, DL2 (first order)	DL2-1	0.892	DL2-2	0.862				
Social-emotional literacy, DL3 (first order)	DL3-1	0.916	DL3-2	0.740				
Digital literacy (second order)	DL1	0.945	DL2	0.967	DL3	0.941		
Digital self-efficacy (first order)	DSE1	0.844	DSE2	0.850	DSE3	0.841	DSE4	0.802
	DSE5	0.801						
Behavioral engagement, OE1 (first order)	OE1-1	0.820	OE1-2	0.873	OE1-3	0.818	OE1-4	0.723
Cognitive engagement, OE2 (first order)	OE2-1	0.844	OE2-2	0.891	OE2-3	0.849	OE2-4	0.839
Affective engagement, OE3 (first order)	OE3-1	0.862	OE3-2	0.888	OE3-3	0.873	OE3-4	0.796
Social engagement, OE4 (first order)	OE4-1	0.845	OE4-2	0.890	OE4-3	0.830	OE4-4	0.839
Online engagement (second order)	OE1	0.938	OE2	0.943	OE3	0.921	OE4	0.924
